# Analysis of mRNA Decay Intermediates in Bacillus subtilis 3′ Exoribonuclease and RNA Helicase Mutant Strains

**DOI:** 10.1128/mbio.00400-22

**Published:** 2022-03-21

**Authors:** Shivani Chhabra, Zachary F. Mandell, Bo Liu, Paul Babitzke, David H. Bechhofer

**Affiliations:** a Icahn School of Medicine at Mount Sinai, Department of Pharmacological Sciences, New York, New York, USA; b The Pennsylvania State University, Department of Biochemistry and Molecular Biology, Center for RNA Molecular Biology, University Park, Pennsylvania, USA; National Institute of Child Health and Human Development (NICHD)

**Keywords:** *Bacillus subtilis*, mRNA decay, 3′ exoribonucleases, RNA helicase, PNPase, Term-seq, transcriptomics

## Abstract

The Bacillus subtilis genome encodes four 3′ exoribonucleases: polynucleotide phosphorylase (PNPase), RNase R, RNase PH, and YhaM. Previous work showed that PNPase, encoded by the *pnpA* gene, is the major 3′ exonuclease involved in mRNA turnover; in a *pnpA* deletion strain, numerous mRNA decay intermediates accumulate. Whether B. subtilis mRNA decay occurs in the context of a degradosome complex is controversial. In this study, global mapping of mRNA decay intermediate 3′ ends within coding sequences was performed in strains that were either deleted for or had an inactivating point mutation in the *pnpA* gene. The patterns of 3′-end accumulation in these strains were highly similar, which may have implications for the role of a degradosome in mRNA decay. A comparison with mapped 3′ ends in a strain lacking CshA, the major RNA helicase, indicated that many mRNAs require both PNPase and CshA for efficient decay. Transcriptome sequencing (RNA-seq) analysis of strains lacking RNase R suggested that this enzyme did not play a major role in mRNA turnover in the wild-type strain. Strains were constructed that contained only one of the four known 3′ exoribonucleases. When RNase R was the only 3′ exonuclease present, it was able to degrade a model mRNA efficiently, showing processive decay even through a strong stem-loop structure that inhibits PNPase processivity. Strains containing only RNase PH or only YhaM were also insensitive to this RNA secondary structure, suggesting the existence of another, as-yet-unidentified, 3′ exoribonuclease.

## INTRODUCTION

mRNA turnover is an essential function that is carried out by various endo- and exoribonucleases in different organisms. In Bacillus subtilis, mRNA decay is hypothesized to initiate with an internal cleavage by the endoribonuclease RNase Y (1, 2). The upstream RNA fragment thus generated is degraded processively in the 3′-to-5′ direction primarily by polynucleotide phosphorylase (PNPase), while the downstream RNA fragment is degraded in the 5′-to-3′ direction by RNase J1 (3, 4). In a strain deleted for the *pnpA* gene, which encodes PNPase, mRNA fragments that contain the 5′ end accumulate, in some cases to a high degree (5–7). It is thought that in the absence of PNPase, mRNA cleavage fragments are acted upon by other RNases, which may be unable to degrade RNA as efficiently as PNPase.

The nature of B. subtilis mRNA decay intermediates has been characterized in a few cases, e.g., *rpsO* mRNA (6) and *ermC* mRNA (8). In the current study, a transcriptome sequencing method (called Term-seq) that was first used to map transcription terminators (9, 10) was used to obtain a transcriptomic view of 3′ ends of mRNA decay fragments in the wild-type (WT) strain compared to strains that were deleted for the *pnpA* gene or the *cshA* gene encoding the DEAD box RNA helicase CshA (11). Recently, we found evidence that PNPase and CshA cooperate in the turnover of several monocistronic mRNAs (12). The accumulation of 3′ ends in the Δ*cshA* strain would identify mRNAs that rely on CshA helicase activity for efficient degradation.

Our Term-seq study also included an analysis of a strain that contained an enzymatically inactive point mutant of PNPase, *pnpA*^D493A^ (13). There is an unresolved question of whether PNPase functions in the context of a degradosome complex in B. subtilis (11, 14, 15). If a degradosome complex is the primary mRNA decay apparatus, the complete absence of PNPase protein in the Δ*pnpA* strain could also impact mRNA decay indirectly by altering degradosome architecture and, thereby, the function of other components such as RNase Y and RNase J1. We assumed that the PNPase point mutant, which has a single altered residue in the enzyme active site, would be present in its native conformation and, therefore, have no impact on the degradosome structure.

In addition to PNPase, B. subtilis is known to contain three other 3′ exoribonucleases: RNase PH (16), YhaM (17), and RNase R (18). The primary functions of these other RNases are thought to be in pathways other than mRNA decay. RNase PH, encoded by the *rph* gene, is the main 3′ exonuclease that removes 3′ nucleotides from precursors of B. subtilis tRNAs that have an encoded CCA sequence (19). B. subtilis RNase PH shares 58% identical residues with Escherichia coli RNase PH, so it is likely to have similar activity, and E. coli RNase PH is distributive on non-tRNA substrates, meaning that it does not remain bound to an RNA molecule to degrade it processively. Instead, RNase PH binds to an RNA, removes a few nucleotides, and then releases the RNA and binds to a different RNA (M. P. Deutscher, personal communication). This attribute would be uncharacteristic of a 3′ exonuclease involved in mRNA decay, which requires high processivity to efficiently degrade RNA fragments. The function of YhaM, encoded by the *yhaM* gene, has not been studied in B. subtilis. However, YhaM interacts specifically with DnaC in the B. subtilis replisome (20) and so may play a part in DNA replication. YhaM of Streptococcus pyogenes was shown to trim 3′ ends of mRNA fragments generated by RNase Y (21), which suggests limited processivity.

RNase R, encoded by the *rnr* gene, shares 39% identical and 59% similar amino acid residues with E. coli RNase R, which is an rRNA quality control enzyme (22). Presumably, RNase R of B. subtilis has the same function as that of the E. coli enzyme, and indirect evidence for this comes from a study in which a defect in 16S rRNA 3′-end processing by YqfG was lethal in B. subtilis unless the *rnr* gene was deleted (23). This finding suggested that the absence of YqfG processing triggers 16S rRNA degradation by RNase R. RNase R of E. coli has intrinsic helicase activity such that the enzyme can degrade through RNA secondary structure (24, 25), and this has also been shown *in vitro* for B. subtilis RNase R (18). Thus, if an mRNA was a target of RNase R activity, the enzyme would be efficient at turning over the mRNA. On the other hand, in exponential-phase E. coli cells, 80% of E. coli RNase R protein is associated with ribosomes, and the fraction of RNase R protein that is not associated with ribosomes is degraded rapidly (26). If this is the case in B. subtilis as well, little RNase R would be available for mRNA turnover. Furthermore, a study of 3′-to-5′ exonuclease activity in Streptococcus pyogenes found that RNase R has very limited activity on mRNAs (27). The current study includes a global analysis of mRNA decay in RNase R mutant strains. We also studied the pattern of decay for *slrA*, a model mRNA that is normally degraded by PNPase (28), in B. subtilis strains containing only one of the four known 3′ exonucleases.

## RESULTS AND DISCUSSION

### Global 3′-end mapping.

To begin to probe B. subtilis mRNA decay globally in more detail than has been done previously, Term-seq (9, 10) was used to identify and quantify all 3′ ends across the transcriptome of WT, *pnpA* deletion, *cshA* deletion, and *pnpA*^D493A^ point mutant strains (see Table S1 in the supplemental material) grown in Luria-Bertani (LB) medium to mid-exponential phase. In Term-seq, a unique RNA oligonucleotide is ligated to the free 3′ hydroxyl end of all RNAs that remain after rRNA depletion, to mark the position of steady-state 3′ ends. RNA molecules are then subjected to library preparation and short-read sequencing on the Illumina platform (see Materials and Methods). Sequencing reads that include the junction between the unique oligonucleotide sequence and the cellular RNA sequence mark the 3′ end of an RNA molecule and are computationally identified and extracted from the total pool of reads. This allows both the identification and quantitation of the abundance of all 3′ ends (see below).

10.1128/mbio.00400-22.3 TABLE S1 B. subtilis strains. Download Table S1, DOCX file, 0.01 MB. Copyright © 2022 Chhabra et al. 2022 Chhabra et al. https://creativecommons.org/licenses/by/4.0/ This content is distributed under the terms of the Creative Commons Attribution 4.0 International license.

**FIG 1:**
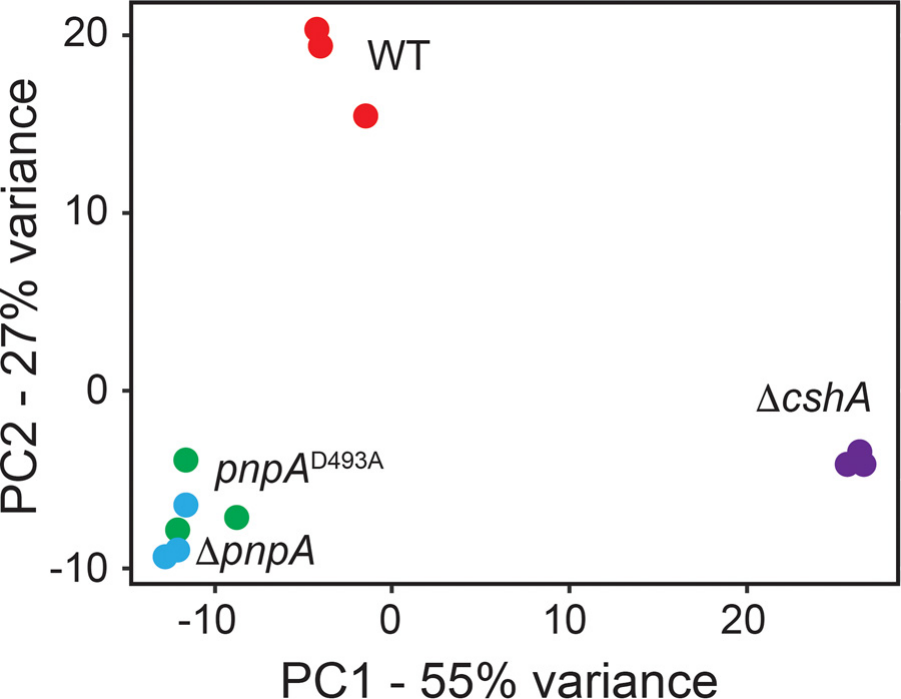
Gene expression profiles cluster by enzymatic availability. A principal-component analysis (PCA) plot of transcriptomics data collected from each Term-seq replicate is shown.

Using the total pool of reads, Term-seq can also yield typical RNA-seq-level information. Thus, Kallisto was used to quantify the raw counts of all transcripts across the B. subtilis transcriptome in each replicate (Table S2). DESeq2 scripts were used to normalize each raw count value via the median-of-ratios method, and a principal-component analysis (PCA) was conducted to compare each inter- and intrastrain replicate (Fig. 1). From this analysis, we observed that the gene expression profiles for the *pnpA* deletion and point mutant strains (Δ*pnpA* and *pnpA*^D493A^) were similar, while the WT strain had a distinct gene expression profile compared to those of the *pnpA* and Δ*cshA* mutant strains.

10.1128/mbio.00400-22.4 TABLE S2 Read abundance (reads/base) for all genes in the wild-type (WT) strain, *pnpA* deletion and point mutant strains, and *cshA* mutant strain. Download Table S2, XLSX file, 0.4 MB. Copyright © 2022 Chhabra et al. 2022 Chhabra et al. https://creativecommons.org/licenses/by/4.0/ This content is distributed under the terms of the Creative Commons Attribution 4.0 International license.

**FIG 2:**
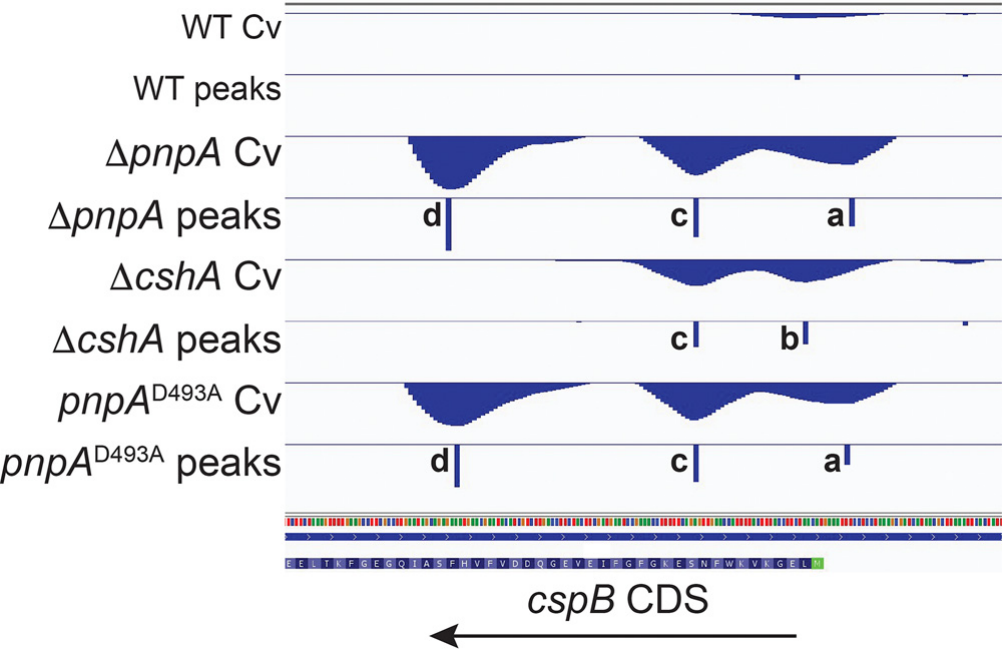
Term-seq 3′-end density across the *cspB* transcript. Shown is an IGV screenshot of the 5′ portion of the *cspB* transcript. Data obtained from each strain are illustrated in two tracks. The top track shows the calculated Cv value at each coordinate, and the bottom track shows the location and height of each identified Cv peak.

To identify and quantify 3′ ends, the first-order derivative of 3′-end read coverage was calculated at each coordinate (see Materials and Methods). These calculations yielded distinct coverage variation (Cv) values for each genomic coordinate. The Cv value at a specific coordinate is correlated with the 3′ abundance at that coordinate; as such, we refer to Cv as 3′-end abundance for the remainder of the manuscript. For each strain, the 3′-end abundance was loaded into the Integrated Genomics Viewer (IGV) in two separate tracks. The example of the *cspB* gene is shown in Fig. 2. The top track for each strain represents the 3′-end abundance value at each coordinate. These 3′-end abundance values appear as “mounds,” with 3′-to-5′ values first increasing toward a peak and then decreasing after the peak. This is the expected pattern for a 3′ exonuclease that encounters an RNA sequence/structure that impedes 3′ exonucleolytic decay. The enzyme “slows down” as it approaches the sequence/structure from the 3′ side, with the accumulation of 3′ ends on the 3′ slope of a mound. The 3′-end abundance peak represents the coordinate with the greatest accumulation of 3′ ends, relative to upstream and downstream nucleotide locations, which occurs at the position where 3′ exonuclease inhibition is the greatest. We postulate that the 5′ side of a mound represents situations in which the inhibitive portion of the transcript is gradually overcome by the 3′ exonuclease, followed by the continuation of processive decay. The lower track for each strain indicates both the coordinate and the relative abundance of all 3′ ends.

For the *cspB* gene in the WT strain, very weak peaks were detected in the 5′ untranslated region (UTR) and near the 5′ end of the coding sequence (CDS) (Fig. 2). The *pnpA* mutant strains showed a peak in the 5′ UTR (Fig. 2, peak a) and in the upstream part of the CDS (Fig. 2, peak c). A peak with an even higher 3′-end abundance (Fig. 2, peak d) was detected further downstream in the CDS. The Δ*cshA* strain shared a peak (Fig. 2, peak c) with the *pnpA* mutant strains and had an additional peak (Fig. 2, peak b) at the upstream end of the CDS. The strong peak that was observed in the *pnpA* mutant strains (Fig. 2, peak d) was not detected in the Δ*cshA* strain. The peak pattern of the *pnpA*^D493A^ strain was similar to that of the Δ*pnpA* strain.

### 3′ ends of mRNA decay intermediates in WT and mutant strains.

**FIG 3:**
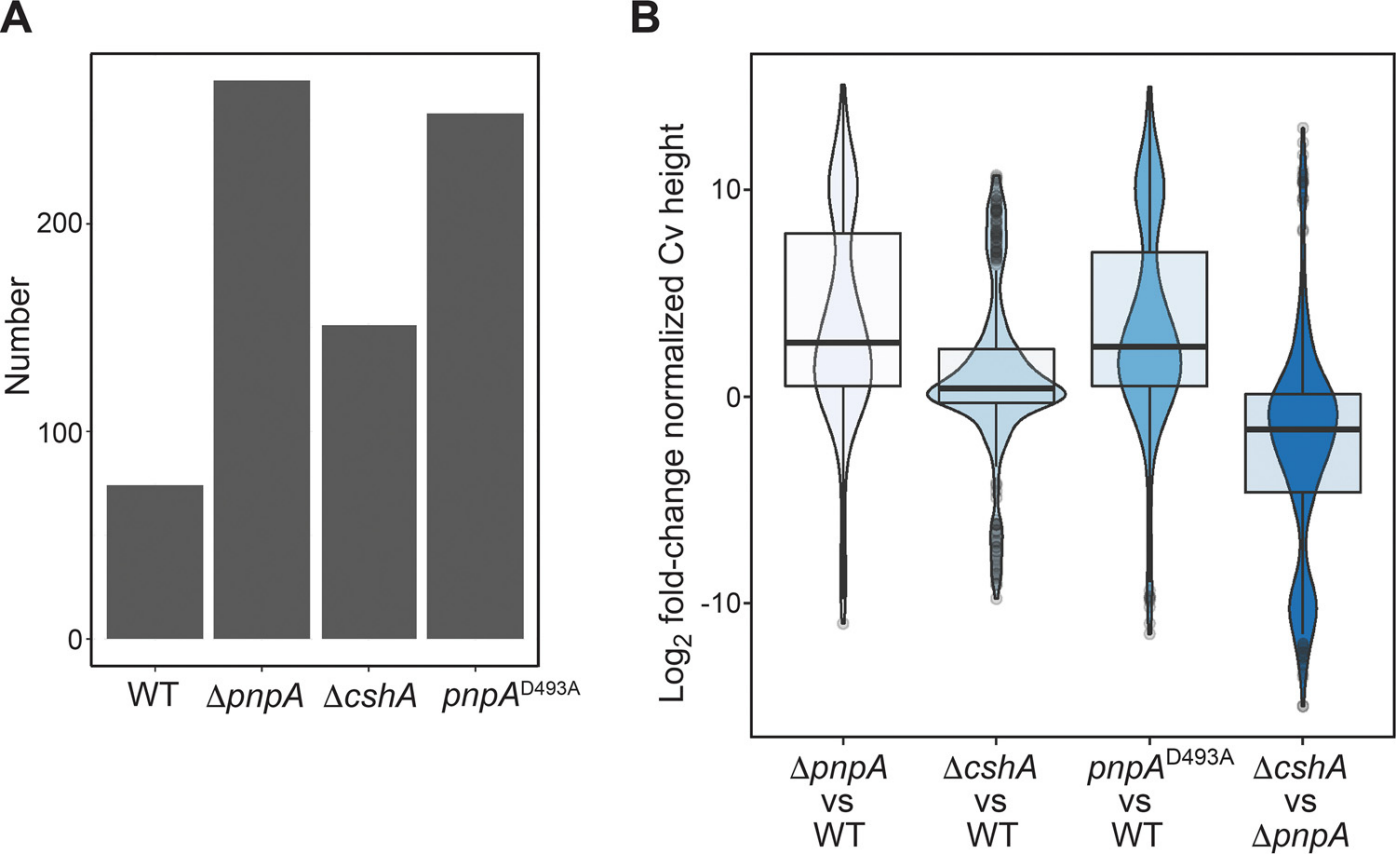
Numbers of monocistronic Cv peaks and Cv peak height increases upon the loss of PNPase or CshA activities. (A) Bar graph showing the number of monocistronic coding sequence Cv peaks identified in each strain. (B) Violin plots overlaid with box plots showing the distribution of the log_2_-normalized Cv peak height fold changes. Each fold change corresponds to the normalized Cv peak height calculated for the strain specified above, divided by the normalized Cv peak height calculated for the strain specified below.

The purpose of 3′-end mapping was to identify mRNA sequences at which 3′ exonucleolytic decay was inhibited. As such, 3′ ends obtained from the Term-seq data set were curated to exclude known transcription terminators (9) and 3′ ends of tRNAs and residual rRNAs. In addition, 3′ ends that mapped to 5′ UTRs were excluded, as these are likely formed from riboswitches or attenuators (10). The remaining mapped 3′ ends were located in CDSs. Furthermore, since the decay of polycistronic mRNA is complex, the analysis was limited to monocistronic genes. B. subtilis contains 987 monocistronic genes, 157 of which were expressed to at least 1 read/base in all strains (Table S3). For this analysis, we included only monocistronic CDS 3′ ends that were found within one of these 157 genes. From this total pool of monocistronic 3′ ends, we took a conservative approach and restricted our initial analysis to 3′ ends with a 3′-end abundance value of 10 or higher in any of the four strains. A comparison of the total numbers of monocistronic 3′ ends identified in each strain that passed our coverage thresholding criteria is shown in the bar graph in Fig. 3A. We observed a 3-fold increase in the number of monocistronic 3′ ends upon the loss of PNPase (Δ*pnpA*) and a 2-fold increase in the number of monocistronic 3′ ends upon the loss of CshA (Δ*cshA*). Of interest was the finding that the number of mapped 3′ ends in the strain with the *pnpA* gene deletion was similar to the number in the strain with the *pnpA* point mutation (*pnpA*^D493A^).

10.1128/mbio.00400-22.5 TABLE S3 Monocistronic CDS with 1-read/base normalized count cutoffs. Download Table S3, XLSX file, 0.02 MB. Copyright © 2022 Chhabra et al. 2022 Chhabra et al. https://creativecommons.org/licenses/by/4.0/ This content is distributed under the terms of the Creative Commons Attribution 4.0 International license.

To construct a singular table of normalized 3′-end abundances, we identified the superset of all 3′ ends presented in Fig. 3A, where 3′ ends that were mapped at positions more than ±5 nucleotides (nt) apart were considered to be different 3′ ends. For each of these 3′ ends, the 3′-end abundance value at that coordinate, divided by the local gene expression upstream of the 3′ end, was calculated using data from each strain. This set of normalized 3′-end abundance values is shown in Table S4. To more accurately determine how the abundance of a particular 3′ end changed upon the loss of PNPase or CshA activity, we calculated the set of log_2_ fold changes of the normalized 3′-end abundance values that were calculated for each strain (Table S4). These log_2_ fold change values were organized into box and violin plots (Fig. 3B). Through this analysis, it was confirmed that the loss of the catalytic activity of PNPase or CshA led to a global increase in the 3′-end abundance. For each of the identified 3′ ends, we also compared the 3′-end abundance in the Δ*cshA* strain to the 3′-end abundance in the Δ*pnpA* strain, where we found that the loss of PNPase had a greater effect on the global 3′-end abundance than the loss of CshA. To further confirm that both PNPase and CshA are involved in global mRNA turnover, we plotted the distribution of normalized 3′-end abundance values for all monocistronic 3′ ends identified in each strain (the sets of 3′ ends shown in Fig. 3A). By comparing each distribution via one-tailed Wilcoxon rank sum exact tests, we found that mutation of either *pnpA* or *cshA* led to a global increase in the 3′-end abundance (Fig. S1). The 3′-end abundance is correlative with the inability of the cellular nucleolytic machinery to degrade a particular 3′ end. Thus, in the absence of PNPase activity, whether due to a complete absence of the protein or a point mutation that inactivates the protein, the remaining 3′ exonuclease activities in the cell do not compensate. In addition, even in the presence of PNPase activity, the absence of CshA helicase activity results in less efficient mRNA turnover.

10.1128/mbio.00400-22.1 FIG S1 PNPase and CshA are involved in global mRNA turnover. Violin plots overlaid with box plots show the distribution of the log_10_-normalized Cv peak heights for all monocistronic Cv peaks identified in each strain. Each line above the plot indicates a statistical comparison between the distributions at each end of the line, with the degree of significance specified by the asterisks above the line. Download FIG S1, TIF file, 1.9 MB. Copyright © 2022 Chhabra et al. 2022 Chhabra et al. https://creativecommons.org/licenses/by/4.0/ This content is distributed under the terms of the Creative Commons Attribution 4.0 International license.

10.1128/mbio.00400-22.6 TABLE S4 Comparison of monocistronic 3′ ends that passed the coverage threshold. Download Table S4, XLSX file, 0.04 MB. Copyright © 2022 Chhabra et al. 2022 Chhabra et al. https://creativecommons.org/licenses/by/4.0/ This content is distributed under the terms of the Creative Commons Attribution 4.0 International license.

To ensure that we were not missing an important role of PNPase and CshA in the processing of 3′ ends that accumulate in 3′ UTRs, we searched for all 3′ ends located between the stop codon and the intrinsic terminator for each of the genes in Table S3 (Table S5). In the WT strain, we identified 22 such 3′ ends. Considering that 88 3′ ends were identified within the CDSs of these genes, we do not find 22 to be an insignificant number. We compared the numbers of 3′ ends that accumulate in 3′ UTRs in each strain (Fig. S2A). Compared to the large change in the numbers of CDS 3′ ends identified when *pnpA* or *cshA* was mutated (Fig. 3A), we found that these changes were minimal in 3′ UTRs. To further quantify the impact of PNPase and CshA on 3′-UTR 3′-end processing, we calculated the normalized 3′-end abundance for each identified 3′-UTR 3′ end and organized these data into violin plots overlaid with box plots (Fig. S2B). While the distribution of normalized 3′-end abundances skewed upward upon mutation of *pnpA* or *cshA*, none of the mutant distributions were statistically different from the WT distribution via one-tailed Wilcoxon rank sum exact tests. We conclude that although there are significant numbers of 3′ ends within 3′ UTRs in the WT strain, neither PNPase nor CshA plays a significant role in processing such 3′ ends.

10.1128/mbio.00400-22.2 FIG S2 PNPase and CshA do not play a significant role in processing 3′ ends that accumulate in 3′ UTRs. (A) Bar graph showing the number of 3′-UTR Cv peaks identified in each strain. (B) Violin plots overlaid with box plots showing the distribution of the log_10_-normalized Cv peak heights for all 3′-UTR Cv peaks identified in each strain. Download FIG S2, TIF file, 2.6 MB. Copyright © 2022 Chhabra et al. 2022 Chhabra et al. https://creativecommons.org/licenses/by/4.0/ This content is distributed under the terms of the Creative Commons Attribution 4.0 International license.

10.1128/mbio.00400-22.7 TABLE S5 3′-UTR 3′ ends identified in each strain. Download Table S5, XLSX file, 0.02 MB. Copyright © 2022 Chhabra et al. 2022 Chhabra et al. https://creativecommons.org/licenses/by/4.0/ This content is distributed under the terms of the Creative Commons Attribution 4.0 International license.

### Overlap of 3′-end mapping in WT and mutant strains.

**FIG 4:**
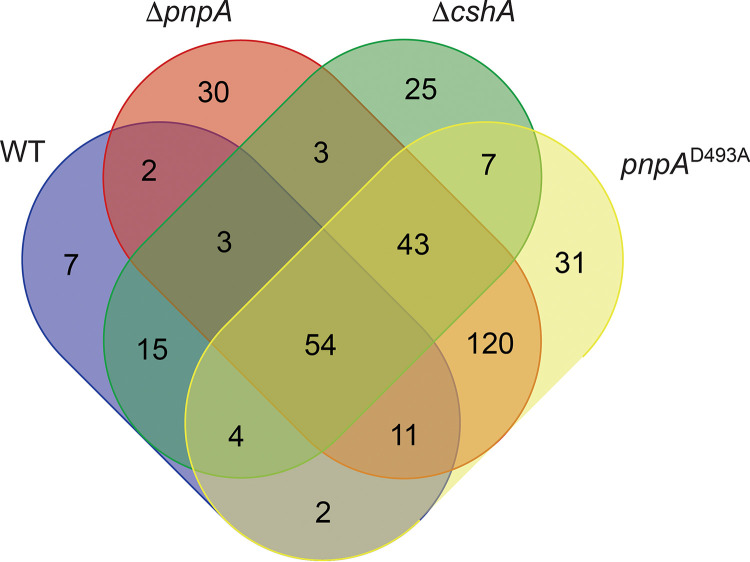
Cv peak overlap between strains. A Venn diagram is shown, where each oval contains the number of monocistronic coding sequence Cv peak locations that were calculated as having a normalized Cv height of ≥0.53 in a particular strain.

A Venn diagram was constructed to determine to what extent the mapped 3′ ends overlapped in the various strains (Fig. 4). In this analysis, a 3′ end was considered to be shared among multiple strains in cases where the coordinate had a normalized 3′-end abundance value of ≥0.53 in all of the compared strains. We chose this minimum value as it was found to be the minimum normalized 3′-end abundance value for the monocistronic 3′ ends identified in the WT strain (Table S4). This approach ensured that a 3′ end was considered to be shared between two strains in situations where there was sufficient 3′-end density at that coordinate in both strains, rather than if the 3′-end peak was identified at that same position in both strains. Through this analysis, it was found that the locations of 3′ ends overlapped considerably between the two *pnpA* mutant strains, with 86% of all 3′ ends identified in the Δ*pnpA* strain also being identified in the *pnpA*^D493A^ strain. The location of mapped 3′ ends in the Δ*cshA* strain also overlapped those in the *pnpA* mutant strains considerably, with 67% of the 3′ ends found in the *cshA* strain also being found in one or both of the *pnpA* strains. These data indicated that, in many cases, both PNPase and CshA activities are required for degradation through sequences that otherwise represent hindrances to 3′-to-5′ decay. This is suggestive of a cooperation of the RNA-unwinding activity of CshA working in advance of PNPase processivity to allow efficient mRNA decay, as was also suggested by previous work (12). On the other hand, ∼60% of the 3′ ends in either of the *pnpA* mutant strains were not shared with the *cshA* mutant strain. Thus, in the absence of PNPase activity, large numbers of 3′ ends accumulate even when CshA helicase activity is present. This finding may indicate that the activity of CshA to unwind mRNA sequences and allow degradation works primarily with PNPase but not with other 3′ exonucleases.

### Relationship between the predicted strength of the RNA secondary structure and mapped 3′ ends.

**FIG 5:**
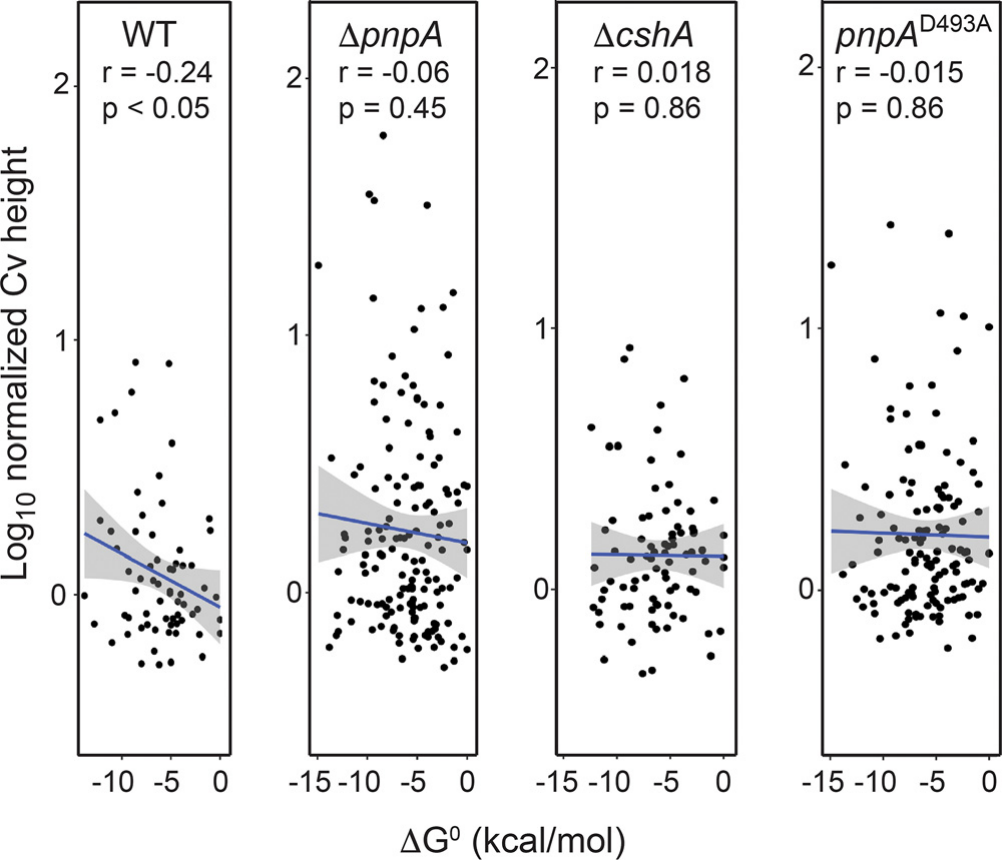
Relationship between the normalized Cv peak height and the upstream minimum free energy of RNA structure. A scatterplot shows the log_10_-transformed normalized Cv peak height and the calculated Δ*G* value for the RNA sequence 40 nt upstream of the identified Cv peak. For each strain, the plot contains all monocistronic coding sequence Cv peaks identified in the WT strain. The result of the linear regression analysis is shown as a blue line, with the standard error shown as gray shading. The strain designation, Spearman correlation analysis *r* value, and *P* value of the linear regression analysis are specified at the top of each plot.

For each strain individually, the log_10_-transformed normalized 3′-end abundance values were plotted against the predicted strength of RNA secondary structure (Δ*G*^0^ value) of the 40 nt upstream of the 3′ end in question, for all coordinates found to have a normalized 3′-end abundance value of ≥0.53 (Fig. 5). We found a weak, yet statistically significant, relationship between the 3′-end abundance and the Δ*G*^0^ of structures identified upstream of the 3′ end in the WT strain. This relationship was abolished in all three mutant strains. Our interpretation of this finding is as follows. PNPase processivity is inhibited by strong secondary structure (29). When PNPase is present, it accesses a free 3′ hydroxyl more rapidly than other 3′ exonucleases and thus is the primary enzyme degrading in the 3′-to-5′ direction. When a secondary structure of considerable strength is encountered, PNPase may stall, giving rise to a 3′ end. We have shown previously that strong secondary structure blocks PNPase processivity, even when CshA is present (12). In the absence of PNPase activity, another 3′ exonuclease, most likely RNase R, is the primary activity degrading in the 3′-to-5′ direction, and it is not sensitive to secondary structure (see the *slrA* results below). Therefore, the presence of RNA secondary structure no longer correlates with 3′-end formation. To explain the lack of a relationship between secondary structure and 3′-end accumulation in the *cshA* strain, we suggest that RNA structures that are less of an obstacle in the WT strain (i.e., less negative Δ*G*) do not hinder PNPase processivity because CshA helicase works with PNPase to allow degradation through such structures. Therefore, only stronger RNA structures with a lower Δ*G* result in mapped 3′ ends. In the *cshA* mutant, however, even weaker structures present an obstacle to PNPase, so there is no relationship between the strength of the predicted structure and the appearance of 3′ ends.

**FIG 6:**
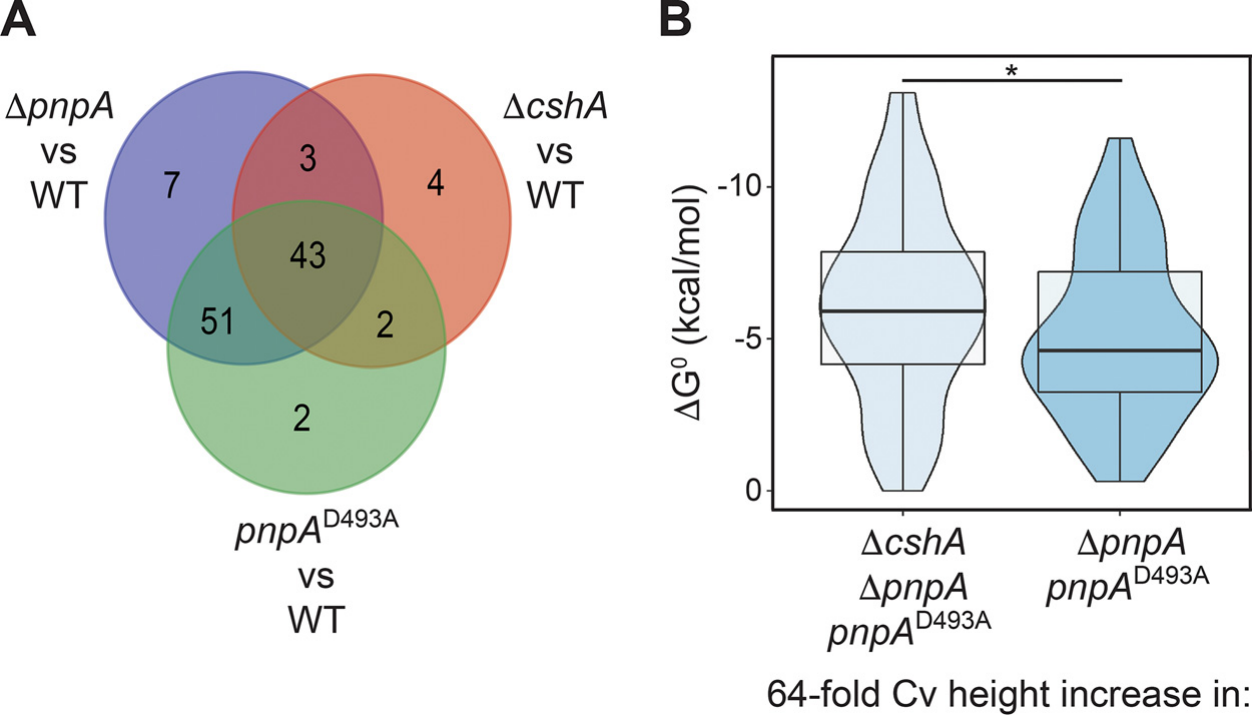
CshA assists PNPase in the degradation of 3′ ends downstream of more stable RNA structures. (A) Venn diagram, where each circle contains the number of monocistronic coding sequence Cv peak locations with a ≥64-fold increase in the normalized Cv height in the specified mutant strain, compared to the WT strain. (B) Violin plots overlaid with box plots showing the distribution of RNA folding minimum free energies for the RNA sequences 40 nt upstream of each Cv peak within the subpopulation specified below the plot. Asterisk indicates p < 0.05 by Wilcoxon Rank Sum Test.

Next, we sought to identify all 3′ ends that significantly increased in abundance upon the loss of PNPase catalytic activity or CshA activity. To do this, we identified all coordinates at which the normalized 3′-end abundance value increased by 64-fold or greater in any of the mutant strains. We chose this strict threshold to ensure that the included 3′ ends had extremely low to no abundance in the WT strain. As such, the 3′ ends in these analyses were efficiently degraded by PNPase in the WT strain and accumulated substantially in the absence of PNPase. From these 3 pools of coordinates, we compiled a second Venn diagram (Fig. 6A). This diagram illustrated that there were two main subpopulations of 3′ ends that experienced buildup in the mutant strains. The first subpopulation consisted of 43 3′ ends that experienced substantial buildup in all three mutant strains; the second subpopulation consisted of 51 3′ ends that experienced substantial buildup only in the two PNPase mutant strains. We compared the Δ*G*^0^ values of the 40 nt upstream of the 3′ ends for both of these subpopulations and found that 3′ ends with significant buildup in all three mutant strains had significantly more stable structures than those that had significant buildup only in the two PNPase mutant strains (Fig. 6B). From these data, we infer that CshA assists PNPase in processively degrading a subpopulation of the total number of 3′ ends upon which PNPase acts. Moreover, CshA is recruited to this subpopulation due to stronger RNA structure upstream of the 3′ ends in question, which PNPase has difficulty degrading on its own.

### Relative location of mapped 3′ ends in CDSs.

**FIG 7:**
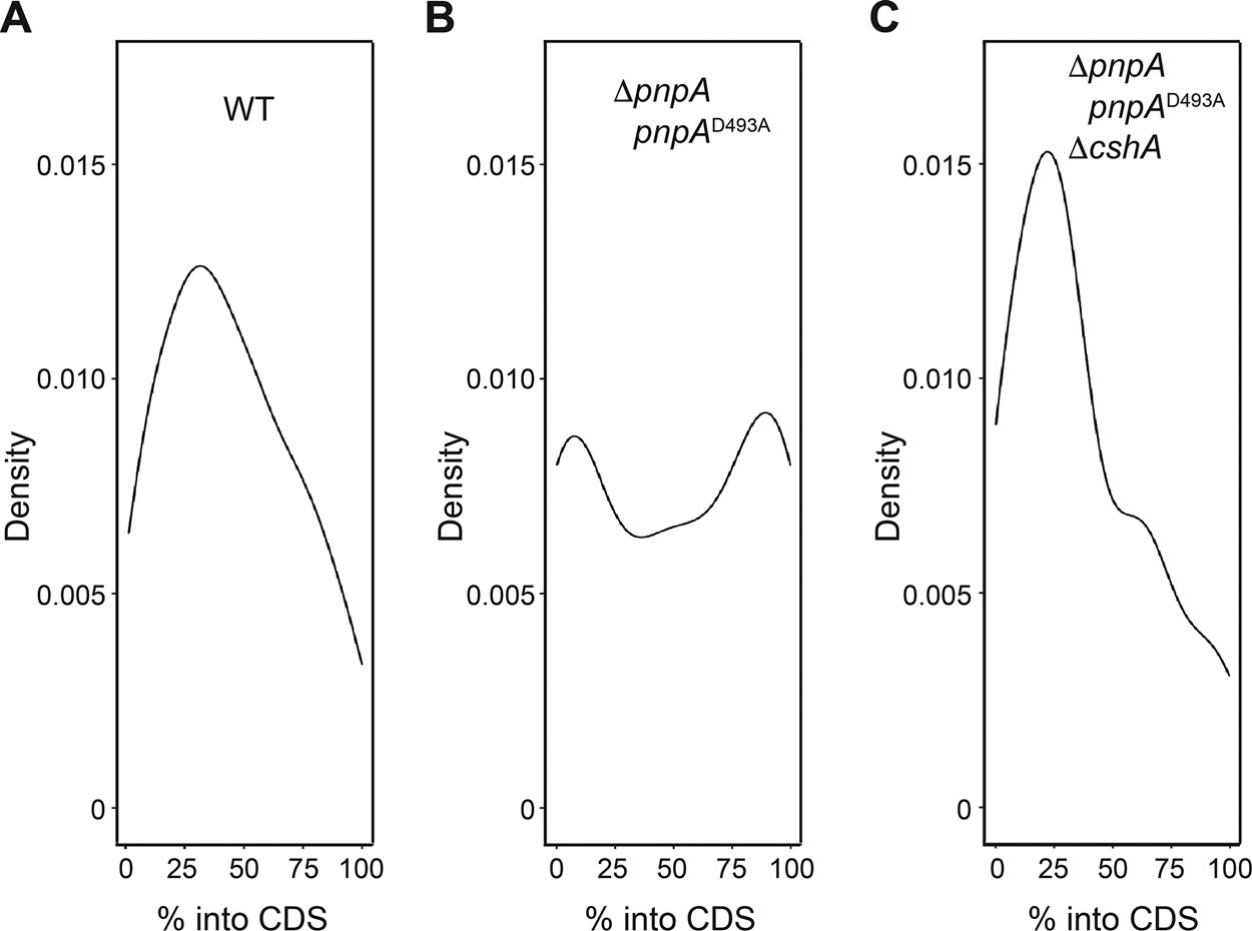
Distribution of Cv peaks across monocistronic coding sequences. (A) Kernel density plot showing the distribution of Cv peaks across the monocistronic coding sequences for all Cv peaks identified in the WT strain. The *y* axis details the approximate probability of finding a Cv peak at a particular location within a monocistronic coding sequence. (B) Same as for panel A except focusing on the distribution of Cv peaks within the subpopulation found to increase in height by 64-fold in the two PNPase mutant strains compared to the WT strain. (C) Same as for panel A except focusing on the distribution of Cv peaks within the subpopulation found to increase in height by 64-fold in all three mutant strains compared to the WT strain.

For the two main 3′ subpopulations referred to in the section above and the 3′ ends identified in the WT strain, density plots were constructed to show the distribution of 3′ ends mapping across CDSs (Fig. 7). As would be expected if decay initiates with internal endonuclease cleavage, 3′ ends would occur predominantly upstream of such cleavage, and this is what was observed in the WT strain where 3′ ends were mapped primarily in the 5′-proximal half of the CDS (Fig. 7A). For peaks that increased in abundance in the PNPase mutant strains only, the distribution was more evenly spread across the CDS (Fig. 7B), which may be explained by WT endonuclease cleavage but poor 3′ exonuclease degradation. Interestingly, the subpopulation that experienced a buildup in all three mutant strains was most concentrated in the first one-third of the CDS (Fig. 7C). This could be due to a higher incidence of structure in the first one-third of CDSs than in the downstream portions of the CDSs. However, a global analysis of RNA structure did not find this to be the case in E. coli (30).

### Summary of Term-seq results.

There are several points that can be made from the Term-seq results. (i) The absence of PNPase activity, whether due to a complete *pnpA* gene deletion or a point mutation in the enzyme active site, leads to an accumulation of mRNA decay intermediates with 3′ ends located in the coding sequence. (ii) The high degree of overlap between mapped 3′ ends in the PNPase deletion mutant and the PNPase point mutant may suggest that mRNA turnover does not occur in the context of a degradosome complex. One could assume that such a complex would be disrupted by the absence of a PNPase trimer (molecular mass of ∼155 kDa) but not affected by the presence in the complex of an inactive PNPase with a point mutation. Since the Term-seq profiles of both *pnpA* mutant strains are very similar, we conclude that a degradosome complex, if it exists, is not crucial for mRNA turnover. (iii) The absence of CshA RNA helicase activity also leads to an accumulation of mapped 3′ ends in coding sequences although to a lesser degree than in the absence of PNPase. About two-thirds of the 3′ ends observed in the *cshA* mutant are also observed in the *pnpA* mutants, indicating that both PNPase and RNA helicase activities are required for the efficient degradation of these mRNAs. (iv) There are many sites in mRNAs that are hindrances to mRNA decay in the absence of PNPase activity, even though CshA is present. This observation may suggest that CshA works primarily with PNPase. There is published evidence for a PNPase-CshA complex *in vivo* (11). On the other hand, the lack of a strong correlation between predicted secondary structures and mapped 3′ ends (Fig. 6) suggests that other factors (e.g., RNA-bound protein or ribosome flow in the opposite direction) may be as important as RNA structure in hindering 3′ exonuclease processivity (11).

### RNase R involvement in mRNA turnover.

In addition to the accumulation of mRNA decay intermediates, a Δ*pnpA* strain shows several phenotypes, including reduced competence and cold sensitivity (31), tetracycline sensitivity (32), and growth in chains (33). The latter phenotype has been attributed to increased expression of the *slrA* gene (28) (see below). On the other hand, the Δ*pnpA* strain grows relatively well, with only a slight decrease in the growth rate (6), suggesting that any effect of the loss of PNPase on mRNA turnover is compensated for by one or more of the other 3′ exoribonucleases. As explained in the introduction, the characteristics of RNase PH and YhaM suggest that they are not involved extensively in mRNA turnover. Thus, we wished to explore further the role that RNase R might play in mRNA decay.

**FIG 8:**
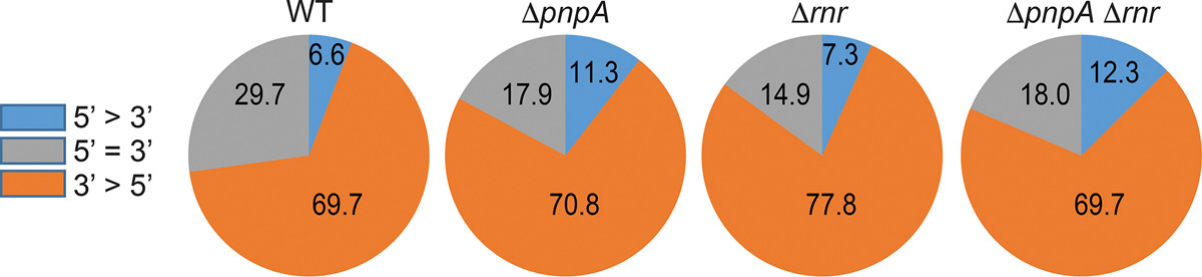
Involvement of RNase R in mRNA decay. Pie charts show the percentage of mRNAs with differential read counts in the 5′ and 3′ one-third of each expressed CDS in WT and RNase mutant strains.

To assess whether RNase R contributes to B. subtilis mRNA turnover, the transcriptomes of WT, Δ*pnpA*, Δ*rnr*, and Δ*pnpA* Δ*rnr* strains were analyzed by RNA-seq. Total RNA was isolated from triplicate cultures of WT and RNase mutant strains growing exponentially in LB medium. RNA-seq library construction, sequencing, mapping to the B. subtilis genome, and normalization of reads were performed as described previously (7). We categorized genes according to the relative read level for the 5′ one-third of the CDS versus the 3′ one-third of the CDS (Table S6). The analysis was based on the hypothesis that a defect in mRNA turnover caused by the absence of a 3′ exoribonuclease leads to an accumulation of 5′-proximal sequences, as is the case in a strain that is missing PNPase (7). CDS reads were divided into three categories: 5′=3′, where the level of reads was uniform across the CDS; 5′-up, where the level of reads in the 5′ one-third of the CDS was ≥1.5-fold higher than that in the 3′ one-third; and 3′-up, where the level of reads in the 3′ one-third of the CDS was ≥1.5-fold higher than that in the 5′ one-third. The percentage of RNAs in each category is shown graphically in Fig. 8. In the WT strain, 6.6% of genes were in the 5′-up category, whereas in the Δ*pnpA* strain, this figure rose to 11.3%. The Δ*rnr* strain was similar to the WT strain, with 7.3% of genes in the 5′-up category. Importantly, a Δ*pnpA* Δ*rnr* double mutant had almost the same percentages of RNAs in all three categories as did the Δ*pnpA* mutant alone. If RNase R could compensate for the absence of PNPase, we would expect that the absence of RNase R in a Δ*pnpA* mutant would lead to a greater number of RNAs in the 5′-up category.

10.1128/mbio.00400-22.8 TABLE S6 Expression profile and ratio-of-ratios (RR) data from the WT and RNase mutants. Download Table S6, XLSX file, 0.3 MB. Copyright © 2022 Chhabra et al. 2022 Chhabra et al. https://creativecommons.org/licenses/by/4.0/ This content is distributed under the terms of the Creative Commons Attribution 4.0 International license.

A more sensitive measure of mRNA decay is obtained by determining the ratio of the 5′ one-third/3′ one-third of reads in the mutant strain, relative to this ratio in the WT strain, or what we have called the ratio of ratios (RR) (7). This measurement is based on the model that decay initiates with endonuclease cleavage, which generates a 3′ hydroxyl that is acted upon by a 3′ exonuclease. Genes with an RR of >1.5 are likely those whose decay from a 3′ end created by endonuclease cleavage requires the activity of the missing 3′ exonuclease. Pairwise Pearson’s correlation of the RR values for the Δ*pnpA*, Δ*rnr*, and Δ*pnpA* Δ*rnr* strains resulted in an *r* value for the Δ*pnpA* and Δ*pnpA* Δ*rnr* strains of 0.878, compared to an *r* value for the Δ*rnr* and Δ*pnpA* Δ*rnr* strains of 0.08. In other words, genes with an elevated RR in the Δ*pnpA* Δ*rnr* double mutant were likely to have an elevated RR in the Δ*pnpA* single mutant as well, whereas genes with an elevated RR value in the Δ*pnpA* Δ*rnr* strain were not likely to have an elevated RR in the Δ*rnr* strain. These results suggested that RNase R does not participate in mRNA decay to a large extent.

### Degradation of *slrA* mRNA in mutant strains containing only PNPase or only RNase R.

**FIG 9:**
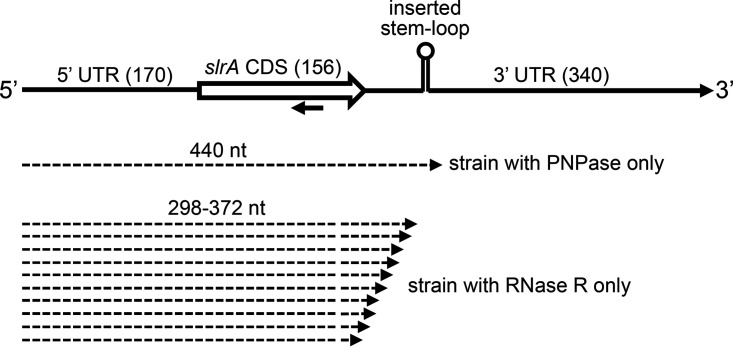
Schematic diagram of the *slrA* gene and 3′-end mapping of prominent decay intermediates. The top line shows segments that comprise the *slrA* gene, with the number of base pairs indicated in parentheses. The strong stem-loop structure, located 60 bp downstream of the CDS, is shown. The oligonucleotide probe used for Northern blotting is indicated by the leftward-facing arrow. Dashed arrows below the gene diagram represent the lengths of prominent decay intermediates in the indicated strains.

To examine mRNA decay in the absence of known redundancy in 3′-to-5′ exonucleolytic activity, we constructed strains that contained only one of the four known 3′ exoribonucleases (Table S1). Gene knockouts of three out of the four 3′ exoribonuclease genes were made with antibiotic resistance marker replacement of all or part of the RNase coding sequences. A model mRNA, encoded by the *slrA* gene, was then used to analyze decay intermediates. The *slrA* gene codes for a regulatory protein that is involved in the expression of the *fla*-*che* operon (34). Termination of *slrA* transcription occurs in a Rho-dependent fashion, resulting in an mRNA with a 3′ terminus that is susceptible to decay by PNPase. We have shown that the short half-life (<1 min) of *slrA* mRNA is dependent on PNPase (28). The ∼670-nt *slrA* mRNA has a relatively long, 340-nt 3′ UTR (Fig. 9). We found previously that the insertion of a strong stem-loop structure (Δ*G* = −18.8 kcal/mol) 60 nt downstream of the *slrA* CDS resulted in the accumulation of an mRNA decay intermediate of ∼440 nt, which was due to hindrance of PNPase-mediated decay by the inserted stem-loop structure (12).

**FIG 10:**
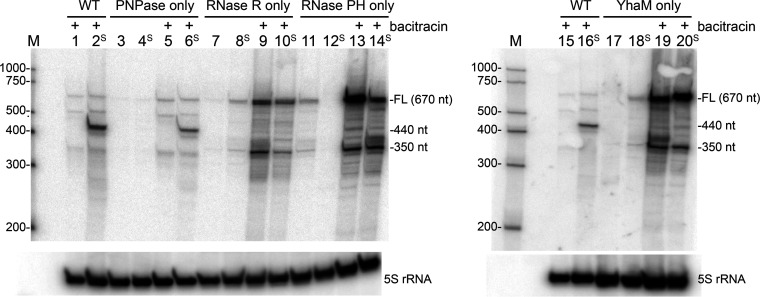
Northern blot analysis of *slrA* decay intermediates. Total RNA was isolated from B. subtilis mutant strains containing only one of four 3′ exoribonuclease activities, as indicated at the top, with or without bacitracin induction. Lanes with “S” indicate the *slrA* construct with the strong stem-loop structure inserted downstream of the *slrA* CDS. The 5S rRNA loading control is shown at the bottom. The marker lane (M) contained RNA Century-Plus markers, with the RNA sizes in nucleotides indicated at the left. Shown are data from one of two biological repeats. FL, full length.

We integrated at the *amyE* locus of each triple RNase deletion strain either a WT copy of the *slrA* gene or a copy of an *slrA* gene construct that had the strong stem-loop structure in the 3′ UTR. Since we found previously that the overexpression of *slrA* causes a severe chaining phenotype (12), *slrA* in the *amyE* locus was under the control of a bacitracin-inducible promoter (3, 35). In the absence of bacitracin, the transcription of the *slrA* construct is completely off, whereas the addition of bacitracin results in the rapid induction of transcription. These strains also contained a copy of the *slrA* gene at its native locus, but the expression of the endogenous *slrA* gene was at such a low level that it did not interfere with the analysis of mRNA transcribed from the bacitracin-inducible construct (Fig. 10, cf. lanes without bacitracin induction).

Bacterial cultures were grown to mid-exponential phase and then induced with bacitracin, as described in Materials and Methods. Total RNA was isolated and analyzed by Northern blotting using an oligonucleotide probe that was complementary to a sequence in the *slrA* CDS (Fig. 10). In the RNase WT strain (i.e., containing WT genes for all four 3′ exonucleases), only minor decay intermediates were detected for the WT *slrA* gene (Fig. 10, lane 1), but a prominent 440-nt decay intermediate was observed in the strain that expressed the *slrA* gene with the strong stem-loop structure inserted into the 3′ UTR (Fig. 10, lane 2). The size of this decay intermediate was consistent with inhibition of 3′-to-5′ exonucleolytic decay at the base of the strong stem-loop structure.

In the strain that contained PNPase only, the full-length *slrA* transcript was present at about the same low level as that in the WT strain, indicating that PNPase is the primary RNase that degrades *slrA* mRNA, as we have observed previously (28). The prominent 440-nt decay intermediate that was observed in the WT strain was also observed in the strain containing PNPase only (Fig. 10, lane 6). RNA isolated from the strain containing only PNPase was analyzed by 3′ rapid amplification of cDNA ends (RACE) to map the 3′ end of the 440-nt decay intermediate. Eight out of nine clones had a 3′ end at precisely the same site, which was 12 nt downstream of the stem-loop structure. Only one clone differed slightly, with a 3′ end that was 3 nt further upstream.

In the strain containing RNase R only, the amount of full-length *slrA* mRNA increased about 2-fold relative to that of the WT strain (Fig. 10, compare lanes 9 and 10 versus lanes 1, 2, 5, and 6), suggesting that RNase R is not as efficient as PNPase in initiating the decay of *slrA* mRNA. In general, the indication is that when PNPase is present, it is more efficient than other 3′ exonucleases in the cell at binding and initiating the processive decay of mRNA fragments.

In the strain that contained RNase R only, we also observed that the prominent 440-nt decay intermediate was no longer detectable (Fig. 10, lane 10). These results indicated that when PNPase was not present, RNase R was able to degrade *slrA* mRNA without the accumulation of the intermediate that represented the inhibition of 3′ exonuclease activity. We postulate that the intrinsic helicase activity of RNase R eliminates the barrier to 3′-to-5′ exonucleolytic decay that the stem structure presents otherwise. When PNPase is present, however, it accesses the mRNA first, and the single-stranded tail downstream of the stem structure that is left when PNPase is inhibited is not long enough for RNase R to bind and initiate decay. In previous work with partially purified B. subtilis protein extracts, we found that an activity attributed to RNase R could not degrade a substrate that had a 12-nt single-stranded extension downstream of a strong stem-loop structure, although it could digest an identical substrate that had a 28-nt extension (18).

While the 440-nt RNA, which arose from a block to PNPase processivity at the strong stem-loop structure, was no longer detected in the strain containing RNase R only (Fig. 10, lane 10), the *slrA* probe detected a shorter RNA of ∼350 nt for both WT and strong-stem *slrA* constructs (Fig. 10, lanes 9 and 10). The size of this RNA was consistent with a fragment whose 3′ end was at or near the end of the CDS. 3′-RACE mapping of the ∼350-nt decay intermediate from the strain containing RNase R only yielded 15 clones with a range of 3′ ends, starting ∼30 nt upstream of the *slrA* stop codon and extending to ∼30 nt downstream of the stop codon (Fig. 9). We hypothesize that RNase R-mediated decay is not sensitive to RNA secondary structure due to its intrinsic helicase activity, but it is sensitive to the presence of ribosomes that are flowing in the 5′-to-3′ direction. Since RNase R is stably associated with ribosomes (26), one can imagine that the binding of RNase R to an mRNA molecule that it is degrading processively in the 3′-to-5′ direction is disrupted when it encounters a ribosome moving on the mRNA in the 5′-to-3′ direction, as the ribosome is then competing for binding to RNase R. As such, RNase R would not be an ideal enzyme responsible for rapid mRNA decay that is characteristic of bacteria. On the other hand, PNPase, which does not have intrinsic helicase activity and which may not always benefit from the associated helicase function, is sensitive to RNA secondary structure (29, 36). We speculate that such sensitivity is important for the protection of mRNAs from the initiation of decay from the 3′ terminus due to the presence of the transcription terminator structure. Instead, decay initiation requires internal cleavage by RNase Y, a step that is perhaps better regulated (37).

### Degradation of *slrA* mRNA in mutant strains containing only RNase PH or only YhaM.

Finally, we constructed strains that contained RNase PH only or YhaM only. In these strains, there was an ∼8-fold increase in the full-length *slrA* mRNA (Fig. 10, lanes 13, 14, 19, and 20), indicating, as we surmised (see the introduction), that these 3′ exoribonucleases are not able to degrade processively from an available 3′ end. Given that decay from the *slrA* 3′ end was not efficient in these strains, it was surprising to detect an increased amount (relative to the WT and PNPase-only strains) of the ∼350-nt RNA. This result suggested that another 3′ exonuclease was degrading *slrA* mRNA. If that was the case, then the paucity of the 440-nt decay intermediate in these strains suggested that this RNase is not sensitive to RNA secondary structure or can work with an RNA helicase. These results hint at yet another 3′ exoribonuclease in B. subtilis. Preliminary work to isolate and characterize such an enzyme is in progress.

## MATERIALS AND METHODS

### Term-seq library generation.

Term-seq was conducted as described previously (9). Total RNA was isolated from three independent cultures of WT, Δ*pnpA*, Δ*cshA*, and *pnpA*^D493A^ strains (see Table S1 in the supplemental material). Cultures were grown overnight at 37°C in LB medium in the presence of antibiotic selection. A total of 100 to 150 μL of the culture grown overnight was used to inoculate 10 mL of LB medium, which was grown without antibiotic selection to mid-exponential phase (80 Klett units). Nine milliliters of the culture was harvested, and total RNA was prepared using the RNeasy minikit (Qiagen), according to the manufacturer’s instructions. Twenty micrograms of total RNA was treated with calf intestinal alkaline phosphatase (CIP) (Quick CIP; New England BioLabs [NEB]). The RNA concentration and quality were checked in a Qubit fluorometer (Thermo Fisher) and a bioanalyzer (Agilent Technologies). All samples had an RNA integrity number (RIN) of ≥9.6. Total RNA was depleted of rRNA using the MICROBExpress bacterial mRNA enrichment kit (Ambion) according to the manufacturer’s instructions and analyzed on a bioanalyzer. rRNA-depleted samples with ≤11% rRNA contamination were ligated with T4 RNA ligase 1 (New England BioLabs) to a unique RNA oligonucleotide (Integrated DNA Technologies) that contained a phosphorylated 5′ end and a 2′,3′ dideoxy 3′ end (5′-pAAUGAGACACUGAGAUCCAGUCGAUGAGCUAddC-3′). RNA samples were submitted to Genewiz for library preparation (NEBNext Ultra II RNA library prep) and paired-end (PE) Illumina sequencing on the HiSeq 4000 platform. Sequence reads were 150 nt, and the Spearman correlation between triplicate samples was >0.97.

### Term-seq data processing.

Custom scripts to process Term-seq data can be found at https://github.com/zfmandell. Term-seq data were processed as described previously (9). After demultiplexing, Illumina adapters were trimmed with Trimmomatic, resulting in a traditional RNA-seq data set (38). From this RNA-seq data set, cutadapt was used to extract all reads that contained the unique RNA oligonucleotide, resulting in the Term-seq data set (39). These Term-seq data sets were mapped separately to the B. subtilis 168 chromosome (GenBank accession number NC_000964.3) via bwa-mem in single-end (SE) mode (40). At this stage, bam files for each pair of replicates were merged. The following steps were applied to both the merged and the nonmerged data sets. Each bam file was split by strand using samtools, and coverage files were generated for each strand-specific bam file using bedtools (41, 42). To identify the comprehensive set of 3′ ends, a series of custom python scripts (found at the GitHub URL mentioned above) was applied to the strand-specific Term-seq coverage files. First, the coverage variation (Cv) was calculated at each nucleotide of each strand-specific coverage file, and the sets of Cv local maxima (peaks) for each file were identified as described previously (9). The Cv magnitude at a 3′ end is tightly correlated with 3′ abundance, which is a function of transcript abundance and 3′-end stability. To limit 3′ ends that could be attributed to noise, only Cv peaks considered with a height of 10 or above were retained, thereby generating the final 3′-end bedgraph files. From this list of 3′ ends, only 3′ ends within monocistronic coding sequences of genes that were found to have ≥1 read per base mapping to the coding sequence were retained for the analysis conducted in this work. All post-adapter-trimming RNA-seq replicate data sets were pseudomapped using Salmon in PE mode with the –validateMappings –seqBias –gcBias options to determine the estimated number of reads, which were normalized using the DESeq2 model (median-of-ratios method) to determine the reads per base (43, 44). To normalize a 3′ end by local upstream expression, the raw Cv height was divided by the log_10_-transformed median RNA-seq coverage in the 50 nt upstream of the 3′ end. The per-base RNA-seq coverage was calculated genome-wide using the bedtools suite (42). The absolute minimum normalized Cv height value was set to be 0.001. To obtain the minimum free energy of RNA folding, the 40 nt upstream of the 3′ end were sent through the *in silico* RNA folding program RNAStructure (45), with default parameters (Table S4).

To conduct a replicate reproducibility analysis, all posttrimming RNA-seq replicate data sets were pseudomapped using Kallisto in SE mode using the –rf-stranded option to a transcriptome built with Illumina-generated RNA-seq data collected from strain PLBS338 (46, 47). This method determined raw count values for each annotated transcript (Table S2). After normalization of the raw count data via DESeq2 models (44), a variance-stabilizing transformation was applied to the data collected from each replicate, and this matrix was projected onto two-dimensional (2-D) space via a principal-component analysis.

### Northern blotting.

For Northern blotting, *slrA* strains were grown in 20 mL LB medium as described above. When the culture reached mid-exponential phase (80 Klett units), 9 mL of the culture was harvested and used as the no-induction control. To the rest of the culture, an aliquot of a freshly prepared bacitracin solution (Sigma-Aldrich) was added to a final concentration of 50 μg/mL. After 20 min of induction, 9 mL of the culture was harvested, and total RNA was extracted by the hot phenol method (48). Twenty micrograms of RNA was loaded per lane on 6% denaturing polyacrylamide gels. The gels were electroblotted and probed for *slrA* RNA using a 5′-end-labeled oligonucleotide that was complementary to *slrA* CDS nt 88 to 119, as described previously (12). Membranes were stripped and reprobed for 5S rRNA, as described previously (49).

### Data availability.

The raw and processed Term-seq data for each replicate can be found in the GEO database under accession number GSE192670.
