# Supplementary material for: Analysis of mRNA Decay Intermediates in Bacillus subtilis 3′ Exoribonuclease and RNA Helicase Mutant Strains
Source: mBio. 2022 Mar 21;13(2):e00400-22. doi: 10.1128/mbio.00400-22 (PMC9040804; doi:10.1128/mbio.00400-22)
Supplement: TABLE S1 [file mbio.00400-22-st001.docx]

Table S1. *B. subtilis* strains.a

| Strain designation | Relevant genotype | 3’ exoribonuclease activity missingc | Source or reference |
| --- | --- | --- | --- |
| BG1 | *trpC2 thr-5* | n/a (wild type) | *B. subtilis* 168 derivative |
| BG546 | *pnpA::kan* | PNPase | (7) |
| BG664 | *rnr::tet* | RNase R | This study |
| BG838 | *cshA::kan* | n/a (CshA missing) | (11) |
| BG900 | *pnpA::kan rnr::spc* | PNPase, RNase R | This study |
| BG1030 | *pnpA*D493A | PNPase | (13) |
| BG1320 | *rnr::tet rph::spc yhaM::phleo amyE::slrA-cat* | RNase R, RNase PH, YhaM (PNPase present) | This study |
| BG1321b | *rnr::tet rph::spc yhaM::phleo amyE::slrA SS-cat* | RNase R, RNase PH, YhaM (PNPase present) | This study |
| BG1322 | *pnpA::kan rph::spc yhaM::phleo amyE::slrA-cat* | PNPase, RNase PH, YhaM (RNase R present) | This study |
| BG1323 | *pnpA::kan rph::spc yhaM::phleo*  *amyE::slrA SS-cat* | PNPase, RNase PH, YhaM  (RNase R present) | This study |
| BG1330 | *pnpA::kan rnr::spc yhaM::phleo amyE::slrA-cat* | PNPase, RNase R, YhaM (RNase PH present) | This study |
| BG1331 | *pnpA::kan rnr::spc yhaM::phleo amyE::slrA SS-cat* | PNPase, RNase R, YhaM (RNase PH present) | This study |
| BG1332 | *pnpA::kan rnr::tet rph::spc amyE::slrA-cat* | PNPase, RNase R, RNase PH (YhaM present) | This study |
| BG1333 | *pnpA::kan rnr::tet rph::spc amyE::slrA SS-cat* | PNPase, RNase R, RNase PH (YhaM present) | This study |

**a** All strains are derivatives of BG1.

b “SS” designation here and below refers to the *slrA* gene with the strong stem-loop structure

downstream of the *slrA* CDS.

C Relevant phenotype added in parentheses
