# Supplementary figures and images for: Analysis of mRNA Decay Intermediates in Bacillus subtilis 3′ Exoribonuclease and RNA Helicase Mutant Strains
Source: mBio. 2022 Mar 21;13(2):e00400-22. doi: 10.1128/mbio.00400-22 (PMC9040804; doi:10.1128/mbio.00400-22)

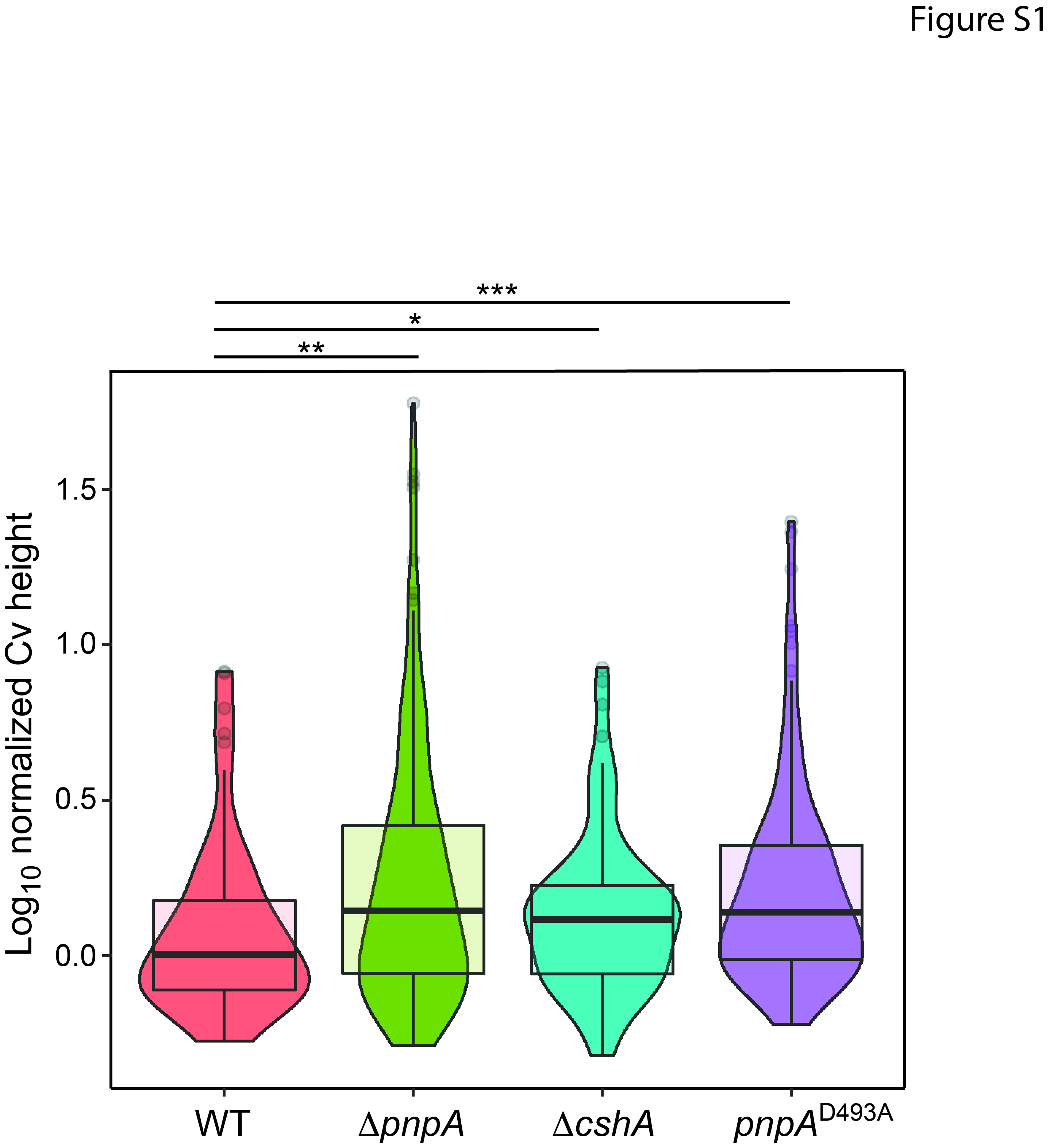

Supplement: FIG S1 [file mbio.00400-22-sf001.tif]

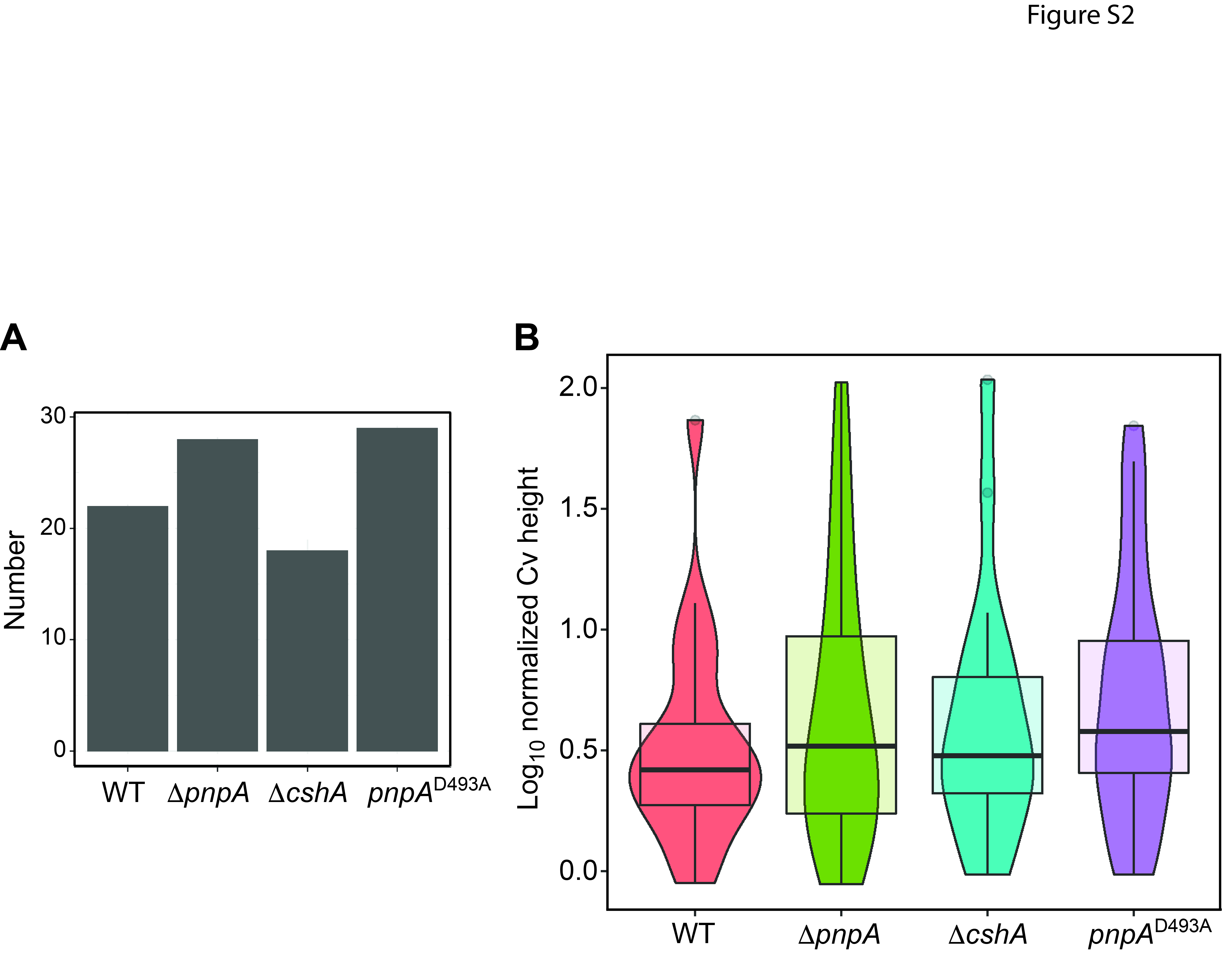

Supplement: FIG S2 [file mbio.00400-22-sf002.tif]
